# Efficacy and Safety of the Subtenon Injection of 0.01% Mitomycin C-augmented Trabeculectomy

**DOI:** 10.7759/cureus.62119

**Published:** 2024-06-10

**Authors:** Rekha R Mudhol, Arkaprava Ray

**Affiliations:** 1 Ophthalmology, Shri BM Patil Medical College, Bijapur Lingayat District Educational Association (Deemed to be University), Vijayapura, IND

**Keywords:** glaucoma-filtering surgery, glaucoma surgery, glaucoma diagnosis and management, glaucoma treatment, ophthalmology

## Abstract

Background

Trabeculectomy, with the application of mitomycin C (MMC), has been the gold standard glaucoma-filtering surgery. The conventional method of applying MMC using soaked sponges does not ensure a controlled amount of delivery of MMC, and incidences of blebitis are reported to be associated with leftover sponges. This study aims to assess the safety and efficacy of a low dose (0.1 mg/ml) of MMC administered through subtenon injection during trabeculectomy combined with cataract extraction, addressing existing research gaps.

Methods

It is a prospective interventional study on patients who underwent trabeculectomy with a subtenon injection of 0.1 mg/ml of MMC combined with cataract extraction and were followed up over six months. Efficacy was determined in terms of intraocular pressure (IOP) reduction; bleb architecture was graded using the Indiana Bleb Appearance Grading System (IBAGS) and success rate, and safety was commented upon in terms of complications.

Results

Thirty patients were enrolled, with the majority having primary open-angle glaucoma (63.33%), while 36.67% had primary angle-closure glaucoma. Baseline IOP was 31.40(±10.38) mmHg. It significantly reduced to 14.60(±3.75) mmHg on the first postoperative day, decreasing to 9.55(±1.57) mmHg by the sixth postoperative month (p=0.001). The percentage reduction in IOP was substantial, 69.57%, by the sixth postoperative visit. Bleb morphology assessment using IBAGS revealed significant improvements in bleb height and extent and a reduction in vascularity over the six-month follow-up (p=0.001), and no eyes had bleb encapsulation. Out of the total patients, 93.33% achieved controlled IOP without anti-glaucoma medications, while 6.67% required one medication for IOP control. Complications were minimal, with transient corneal edema in six patients and manageable postoperative hypotony in one case.

Conclusion

A subtenon injection of MMC during trabeculectomy effectively reduces IOP and promotes favorable bleb architecture, offering an effective and safer alternative to the conventional approach.

## Introduction

Glaucoma is characterized by optic neuropathy, involving specific optic disc anomalies and functional deficits detected through automated visual field testing, with elevated intraocular pressure (IOP) recognized as a risk factor but not a defining feature [1]. Glaucoma now stands as the second leading cause of global blindness, following cataracts, as reported by the World Health Organization [2]. The worldwide prevalence of glaucoma in individuals aged 40-80 years is approximately 3.54% [3]. Primary angle-closure glaucoma (PACG) is more prevalent in Asia at 1.09%, while primary open-angle glaucoma (POAG) is more prevalent in Africa at 4.20%. As of 2013, the global estimate of individuals with glaucoma aged 40-80 was 64.3 million, with projections indicating an increase to over 111.8 million by the year 2040 [3]. Glaucoma management primarily focuses on reducing IOP, with the American Academy of Ophthalmology recommending an initial target of a 25% reduction from baseline in POAG [4-6]. Topical medication is the primary therapy, but issues like tolerability, insufficient IOP reduction, or poor compliance necessitate a shift towards filtering surgeries [7]. Since its inception in the mid-1960s, trabeculectomy has been the gold standard surgery for treating glaucoma [7,8].

Mitomycin C (MMC) is extensively employed in diverse surgeries, such as glaucoma, pterygium, cicatricial eye disease, conjunctival neoplasia, dacryocystorhinostomy, and allergic eye disease [9-11]. In glaucoma surgery, notably trabeculectomy, MMC has been routinely applied for over two decades to mitigate postoperative episcleral fibrosis and prevent bleb failure attributed to scarring [10-12]. Its impact on enhancing fibroblast density and connective tissue improves long-term IOP control in glaucoma-filtering surgery [10-14]. The conventional method of applying MMC is with soaked sponges placed in the subconjunctival space along with adjusting concentration and exposure duration based on the risk of failure [15]. An alternative approach currently under investigation is the intraoperative injection of MMC, serving as an alternative to sponge application [10].

Intraoperative MMC injection in trabeculectomy offers advantages over sponge application, generating diffuse and elevated blebs. This method may enhance long-term success without increased complications, particularly by promoting less scarring and vascularization of the bleb [10,16]. Additionally, the injection approach eliminates the need for multiple sponges, reducing the risk of retained sponges [17-19]. To our knowledge, there is limited research on the intraoperative injection of MMC via the subtenon route, and even fewer studies have investigated the use of a lower dose of 0.1 mg/ml of MMC for intraoperative subtenon injection. To address this gap, our study evaluates the safety and efficacy of administering a low dose (0.1 mg/ml) of MMC through subtenon injection during trabeculectomy.

## Materials and methods

Study design

This prospective interventional study was conducted at the tertiary care hospital in Northern Karnataka, involving a consecutive series of trabeculectomies augmented with MMC combined with cataract extraction over one year from September 2022 to August 2023. Thirty participants meeting the inclusion criteria were enrolled in the study. Included were patients aged over 25 years with conditions such as POAG not adequately controlled by anti-glaucoma medications, PACG, pseudoexfoliation glaucoma, or normal-tension glaucoma not effectively managed by anti-glaucoma medications. Exclusion criteria comprised a history of prior ocular surgery, conjunctival manipulation, and ocular or systemic comorbidities that could influence the procedure and study outcomes, including immunodeficiency, connective tissue disease, and uncontrolled diabetes.

Informed consent was acquired in the vernacular language, in the presence of a witness before enrolling each participant. A thorough preoperative assessment was done, involving a detailed patient history encompassing demographics, present and past ocular and systemic conditions, prior ocular surgeries, family history of glaucoma, and personal habits. A detailed ocular examination was conducted using a slit lamp (AIA-11-5S-L; Appasamy Associates, Chennai, India). Best corrected visual acuity (BCVA) was measured and documented in the logarithm of the minimum angle of resolution (LogMAR). Baseline IOP was measured using the Goldmann applanation tonometer (AATM 5001; Appasamy Associates). Gonioscopy using a four-mirror goniolens (MIPL/14; Opticlear Ophthalmic Lenses) was performed and was graded using Shaffer's anterior chamber angle grading system [20]. Peripheral anterior chamber depth was graded per the van Herick grading system [21]. The visual field was analyzed using a 24-2 Swedish Interactive Threshold Algorithm (SITA) program in a Humphrey field analyzer (740i; Zeiss, Oberkochen, Germany). Fundus examination was conducted by both slit lamp biomicroscopy using a +90D lens (V90C; Volk, Mentor, Ohio, United States) and binocular indirect ophthalmoscopy (AIO-7; Appasamy Associates) and baseline cup-to-disc ratio was documented.

Surgical procedure

**Figure 1:**
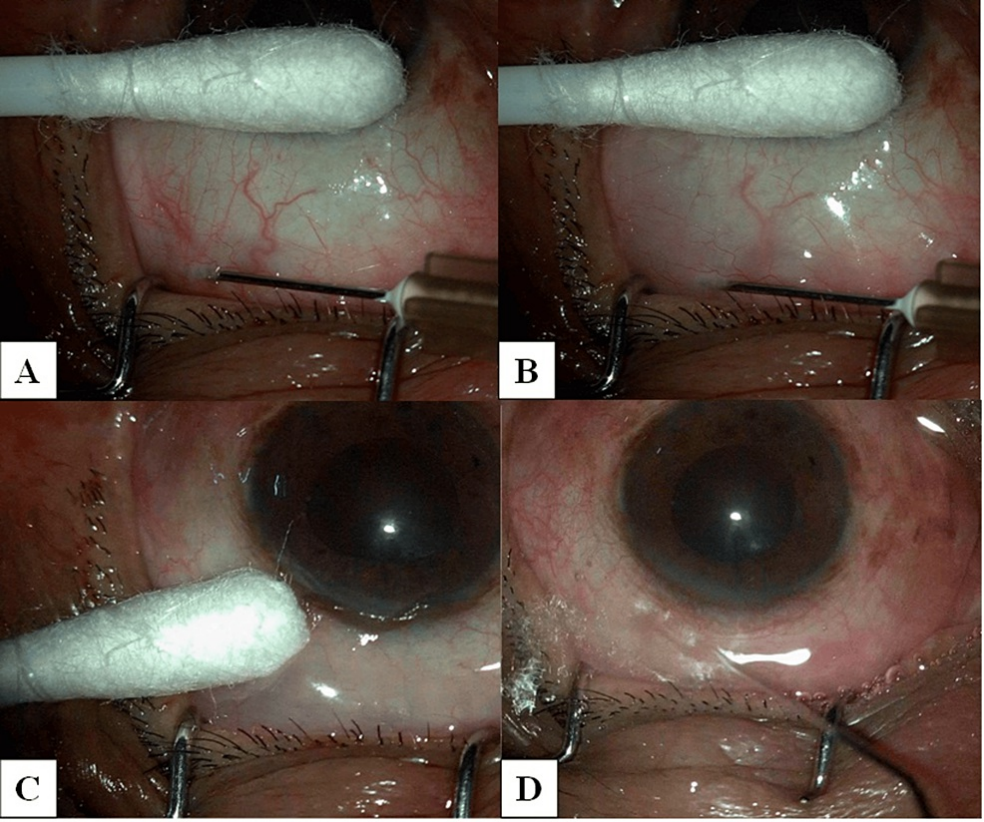
(A) A 26-gauge needle was administered in the subtenon space 8 mm distal to the limbus. (B) 0.1 ml of 0.1 mg/ml of mitomycin C is injected. (C) Massaged away from the limbus using a cotton bud. (D) A thorough wash was given with 30 ml of 0.9% normal saline.

A single experienced surgeon performed all the surgeries. To prepare the MMC solution, we mixed 2 mg MMC with 5 ml of sterile water for injection. We took 0.1 ml of this solution in a 1 ml tuberculin syringe and diluted it with 0.3 ml of 2% lignocaine hydrochloride to make it 0.1 mg/ml of MMC. We discarded 0.3 ml of the solution and considered only 0.1 ml for injection. All patients received intravenous 20% mannitol infusion preoperatively at a dose of 1 g/kg of body weight under blood pressure monitoring. For achieving local anesthesia, a 5 ml solution comprising a 1:1 mixture of 0.5% bupivacaine hydrochloride and 2% lignocaine hydrochloride without adrenaline, along with 5 IU/ml hyaluronidase, was administered into the peribulbar space. Using a 26-gauge needle, 0.1 ml of 0.1 mg/ml of MMC was injected 8 mm distal to the superior limbus and was massaged away from the limbus to spread the MMC (Figure 1).

**Figure 2:**
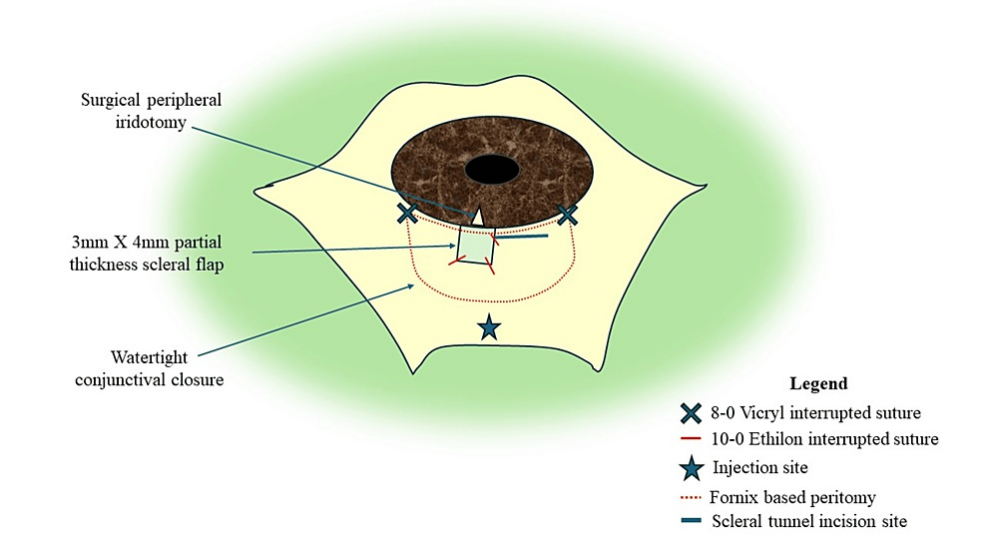
Surgical site architecture showing the site for subtenon injection and scleral flap with the location of sutures used. Image Credit: Arkaprava Ray

Fornix-based conjunctival peritomy followed by a light wet field cautery was done. We raised a 3×4 mm half-thickness scleral flap. A 5.5 mm scleral incision was given adjacent to the scleral flap, a sclerocorneal tunnel was constructed, and conventional small incision cataract surgery was performed along with a rigid poly(methyl methacrylate) (PMMA) posterior chamber intraocular lens implantation (Appalens 209; Appasamy Associates) in the capsular bag. With Kelly's Descemet membrane punch, a 1×1 mm sclerotomy was created under the scleral flap. A peripheral surgical iridectomy was carried out, and subsequently, the scleral flap was repositioned and secured in place with four 10-0 monofilament nylon sutures. After confirming a well-maintained anterior chamber, the conjunctiva was closed using two interrupted 8-0 Vicryl sutures to ensure a watertight seal (Figure 2).

Postoperative evaluation and outcome measurement

Data were collected postoperatively on day 1, week 1, month 1, month 3, and month 6. Visual acuity was measured and quantified in LogMAR. IOP was measured using a Goldmann applanation tonometer (AATM 5001; Appasamy Associates), bleb grading was done using the Indiana Bleb Appearance Grading System (IBAGS), and bleb extent, height, and vascularity were graded in all visits [22]. Anterior chamber depth was assessed using Spaeth's clinical classification of shallow anterior chamber [23]. Documentation of any complications, such as bleb leaks, hypotony (characterized by IOP less than 6 mmHg), infection, and corneal edema/haze, was undertaken.

The criteria for surgical success were clearly defined. Complete success was defined as postoperative IOP less than or equal to 18 mmHg, achieved without the necessity for anti-glaucoma medications or interventions. In cases where additional anti-glaucoma medications were required postoperatively, the outcome was categorized as qualified success. However, if the postoperative IOP exceeded 18 mmHg, even with the administration of additional anti-glaucoma medications, the result was deemed a failure.

Statistical analysis

Descriptive variables were expressed in frequency (percentage) or mean with standard deviation (SD). The association between categorical variables during postoperative follow-ups was assessed using the Friedman test. The Mann-Whitney U test was employed to determine significant differences in IOP reduction between the open-angle and closed-angle groups post-surgery. Bleb parameters over follow-ups were analyzed using Cochran's Q test. A p-value of less than 0.05 was considered statistically significant. The statistical analysis was conducted using IBM SPSS Statistics for Windows, Version 29.0 (Released 2023; IBM Corp., Armonk, New York, United States).

## Results

In this study, a total of 30 patients were enrolled and closely monitored for a period of six months. The participants had a mean age of 66.93 years (±10.06), with a majority of 19 (63.30%) being male and 11 (36.67%) females. Among the enrolled patients, 19 (63.33%) were diagnosed with POAG, and 11 (36.67%) had PACG. The mean baseline visual acuity (measured in LogMAR units) was 1.11(±0.32), while the baseline IOP was 31.40 mmHg (±10.38). The mean cup-to-disc ratio at the initial presentation was 0.75(±0.12) (Table 1).

**Table 1:** Baseline characteristics. LogMAR: logarithm of minimum angle of resolution; SD: standard deviation; n: number of participants

Characteristics	Frequency (n)	Percentage (%)
Mean age in years (±SD)	66.93(±10.062)	
Gender		
Male	19	63.3
Female	11	36.7
Type of glaucoma		
Primary open-angle glaucoma	19	63.33
Primary angle-closure glaucoma	11	36.67
Systemic comorbidities		
Diabetes	2	6.7
Hypertension	5	16.7
Mean baseline parameters		
Mean baseline intraocular pressure in mmHg (±SD)	31.40(±10.38)	
Mean baseline vision in LogMAR (±SD)	1.11(±0.32)	
Mean baseline cup-to-disc ratio (±SD)	0.75(±0.12)	

The mean visual acuity in LogMAR (±SD) at presentation was 1.11(±0.32). On the first postoperative day, it improved to 0.86(±0.26). In the first week, first month, third month, and sixth month, the mean visual acuity was 0.69(±0.30), 0.56(±0.31), 0.51(±0.33), and 0.46(±0.33), respectively. The mean visual acuity significantly improved over the follow-ups, with a p-value of 0.001 estimated using the Friedman test (Table 2).

**Table 2:** Mean visual acuity in LogMAR over follow-ups. *p<0.05 is considered to be statistically significant. SD: standard deviation; LogMAR: logarithm of the minimum angle of resolution

Visual acuity over follow-ups	Mean	SD	Friedman test	P-value
At presentation	1.11	0.32	116.67	0.001***
Postoperative day 1	0.86	0.26		
Postoperative week 1	0.69	0.30		
Postoperative month 1	0.56	0.31		
Postoperative month 3	0.51	0.33		
Postoperative month 6	0.46	0.33		

**Figure 3:**
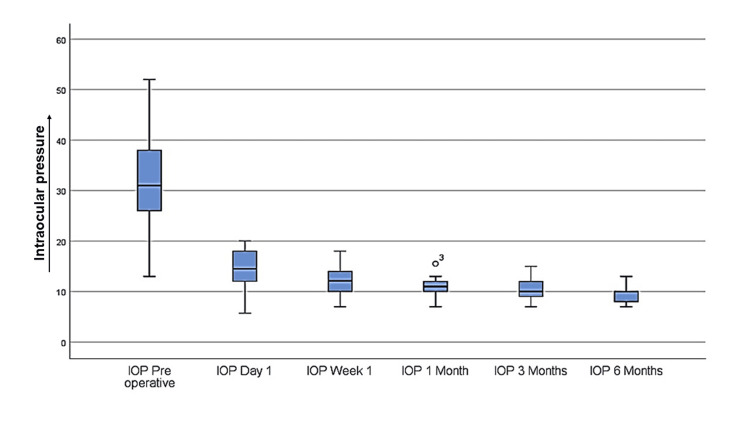
Box and whisker plot showing IOP with its range over all follow-ups. IOP: intraocular pressure

There was a significant reduction in IOP during the postoperative period. At the initial presentation, the mean IOP was 31.40 mmHg (±10.38), which markedly reduced to 14.60 mmHg (±3.75) on the first postoperative day. This reduction continued, reaching 9.55 mmHg (±1.57) by the sixth postoperative month, reflecting a statistically significant trend with a p-value of 0.001. The percentage reduction in IOP was substantial, measuring 53.55% on the first postoperative day and further increasing to 69.57% on the sixth postoperative visit (Table 3; Figure 3).

**Table 3:** Mean intraocular pressure over the follow-ups with percentage reduction. *p<0.05 is considered to be statistically significant. SD: standard deviation

Follow-ups	Mean	SD	Percentage reduction	Friedman test	P-value
At presentation	31.40	10.38	-	104.27	0.001***
Postoperative day 1	14.60	3.75	53.55%		
Postoperative week 1	12.32	2.92	60.74%		
Postoperative month 1	10.89	1.90	65.35%		
Postoperative month 3	10.20	1.98	67.51%		
Postoperative month 6	9.55	1.57	69.57%		

The initial IOP at presentation was markedly higher in patients with closed-angle glaucoma (37.64±7.50 mmHg) compared to those with open-angle glaucoma (27.79±1.26 mmHg), demonstrating statistical significance with a p-value of 0.008. Following surgery, both groups experienced a reduction in IOP, reaching 9.24±1.56 mmHg in the open-angle glaucoma group and 10.09±1.51 mmHg in the closed-angle glaucoma group after six months, having no significant difference in IOP between the two groups (p=0.103). This suggests that while the initial IOP disparity between open-angle and closed-angle glaucoma patients was notable, the surgical interventions effectively equalized IOP levels in both groups over the six-month follow-up period (Table 4).

**Table 4:** Comparison of IOP reduction among open-angle glaucoma and closed-angle glaucoma. *p<0.05 is considered to be statistically significant. SD: standard deviation; IOP: intraocular pressure

IOP	Primary open-angle glaucoma (n=19)		Primary closed-angle glaucoma (n=11)		Mann-Whitney test	P-value
	Mean	SD	Mean	SD		
At presentation	27.79	10.26	37.64	7.50	44.50	0.008**
Postoperative day 1	14.26	3.94	15.18	3.49	88.50	0.497
Postoperative week 1	12.06	2.64	12.76	3.44	88.50	0.497
Postoperative month 1	10.83	2.03	11.00	1.73	96.00	0.735
Postoperative month 3	10.43	1.99	9.81	1.99	89.00	0.525
Postoperative month 6	9.24	1.56	10.09	1.51	66.00	0.103

Over the follow-ups, bleb height increased with two, 16, 24, 28, and 29 patients having high bleb at postoperative day 1, week 1, month 1, month 3, and month 6, respectively (p=0.001). Bleb extent also improved over the follow-up period, with 15, 25, 28, 29, and 29 patients spanning more than two clock hours at postoperative day 1, week 1, month 1, month 3, and month 6, respectively (p=0.001). Vascularity of bleb reduced over time with 30, 28, 20, 12, and 5 at postoperative day 1, week 1, month 1, month 3, and month 6, respectively (p=0.001) (Table 5).

**Table 5:** Bleb characteristics per IBAGS over follow-up. *Cochran's Q test *p<0.05 is considered to be statistically significant. IBAGS: Indiana Bleb Appearance Grading System

Bleb parameters and grading as per IBAGS		Day 1	Week 1	Month 1	Month 3	Month 6	P-value
Height	Low (H_0_,H_1_)	28	14	6	2	1	0.001^***^
	High (H_2_,H_3_)	2	16	24	28	29	
Extent	<2 clock hours (E_0_,E_1_)	15	5	2	1	1	0.001^***^
	>2 clock hours (E_2_,E_3_)	15	25	28	29	29	
Vascularity	Low vascularity (V_0_,V_1_)	0	2	10	18	25	0.001^***^
	High vascularity (V_2_,V_3_)	30	28	20	12	5	

Twenty-eight patients (93.33%) demonstrated controlled IOP according to the pre-defined criteria without needing any anti-glaucoma medications, indicating complete success. Conversely, two patients (6.67%) achieved IOP control with the use of one anti-glaucoma medication, categorizing them as qualified success cases. Importantly, no instances of failure where IOP was uncontrolled were reported in the study (Table 6).

**Table 6:** Distribution of surgical success till the last follow-up.

Surgical outcome	Frequency (n, percentage)
Complete success	28 (93.33%)
Qualified success	2 (6.67%)
Failure	0 (0%)

Postoperatively, corneal edema was observed in six patients; however, it subsided within a few days after the surgical procedure. Additionally, one patient experienced hypotony in the immediate postoperative period, which was effectively managed conservatively without the necessity for surgical intervention. Notably, no complications such as bleb leaks or encapsulations or infections were reported, indicating a favorable and uneventful postoperative course for most patients.

## Discussion

Trabeculectomy, which is the gold standard glaucoma-filtering surgery, has advanced significantly with the use of antimetabolites like MMC. Traditionally, MMC is soaked in sponges and applied in the subconjunctival space. With the conventional route, incidents of blebitis and lost MMC-soaked sponges have been reported, leading to the development of foreign body granulomas [18,19]. To overcome these complications, the subtenon injection of low-dose MMC, which ensures the precise delivery of the desired amount of drug, has been taken up in the present study. This study assesses the safety and efficacy of this approach with a lower dose (0.1 mg/ml) over six months. Thirty patients were enrolled, with the majority having POAG (63.33%), while 36.67% had PACG. Baseline IOP was significantly reduced from 31.40(±10.38) mmHg to 9.55(±1.57) mmHg by the sixth month (p=0.001), achieving a 69.57% reduction. Bleb morphology in terms of height, extent, and vascularity improved significantly over six months, giving diffuse, elevated, and minimally vascular blebs (p=0.001) with no bleb encapsulation. Most patients (93.33%) achieved controlled IOP without additional medications, while 6.67% required one medication. Complications were minimal, with transient corneal edema in six patients and manageable postoperative hypotony in one case.

In our study, the average age of participants was 66.93(±10.06) years (Table 1), aligning with findings from a study conducted in southern India by Senthilkumar et al. [24]. However, it differed from those Rajendrababu et al. reported, with a mean age of 46.41(±20.43) years [25]. Our study enrolled 63.33% of POAG patients and 36.67% of PACG patients (Table 1), similar to the study by Mudhol and Bansal conducted in a similar demography [26]. Within our cohort of enrolled subjects, the mean preoperative IOP was documented at 31.40(±10.38) mmHg (Table 1), which was comparably lower than the study performed by Gupta et al., where the preoperative IOP was reported as 43.05(±10.30) mmHg and can be attributed to there more inclusion of angle-closure glaucoma [27]. 

Our study demonstrates a significant reduction in IOP postoperatively. The mean IOP markedly reduced to 14.60(±3.75) mmHg on the first postoperative day, with a continued decline to 9.55(±1.57) mmHg by the sixth postoperative month. There was a significant initial reduction of 53.55% in IOP on the first postoperative day and 69.57% by six months (Table 3). In contrast to our study, Gupta et al. utilized a 0.02% MMC subtenon injection and observed a reduction in mean IOP to 13.50(±5.65) on the first postoperative day, but it raised to 15.17(±2.48) by the sixth postoperative month [27]. However, their mean percentage reduction in IOP until the last follow-up was comparable to our findings at 68% (Table 3), which can be attributed to their consideration of a higher initial mean IOP. Whereas Maheshwari et al. employed a subconjunctival injection of 0.04% MMC and achieved a significant reduction in IOP from 29.00(±11.92) mmHg preoperatively to 12.00(±6.12) mmHg at the second postoperative week, and this reduction remained stable throughout the 12-month follow-up, with the mean IOP being 12.19(±4.03) mmHg at the last follow-up [10], Shih and Chen demonstrated a two-staged approach of intra-tenon injection of 0.01% MMC, administered four hours before trabeculectomy, and as per their reported outcomes, IOP was reduced from baseline 33.50(±10.10) mmHg to 15.26(±6.08) mmHg at six months follow-up which was statistically significant [28]. Senthilkumar et al. demonstrated twin-site combined phacoemulsification and MMC-augmented trabeculectomy, and they achieved a 24.90% IOP reduction at their last follow-up, which is comparably lower than our findings of 69.57% at the last follow-up (Table 3). However, it can be attributed to their lower baseline IOP considerations (19.80±4.70 mmHg) [24].

We observed a significant discrepancy in IOP at the presentation, with closed-angle glaucoma patients exhibiting markedly higher initial IOP (37.64±7.50 mmHg) compared to those with open-angle glaucoma (27.79±1.26 mmHg). Post-surgery, we observed a substantial reduction in IOP in both groups, reaching 9.24(±1.56) mmHg in the open-angle glaucoma group and 10.09(±1.51) mmHg in the closed-angle glaucoma group after six months. And at six months postoperatively, no significant difference in IOP existed between the two groups, suggesting that although an initial IOP disparity was evident, the surgical interventions effectively equalized IOP levels in both open-angle and closed-angle glaucoma patients over the six-month follow-up period (Table 4). Our finding aligned with the six-month results reported by Maheshwari et al. [29]. However, Maheshwari et al. reported a higher success rate in open-angle glaucoma (68.8%) than in closed-angle glaucoma (55.2%) at 36 months, indicating potential long-term variations in surgical outcomes between both groups [29].

Senthilkumar et al. demonstrated that phacotrabeculectomy augmented with 0.2 mg/dl MMC-soaked sponges had a mean baseline LogMAR BCVA of 0.80(±0.4), which improved to 0.11(±0.20) at six months of follow-up [24]. In our study, the mean baseline LogMAR BCVA was 1.11(±0.32), which improved to 0.46(±0.33) at six months. This difference can be attributed to a higher preoperative cup-to-disc ratio in our patients.

**Figure 4:**
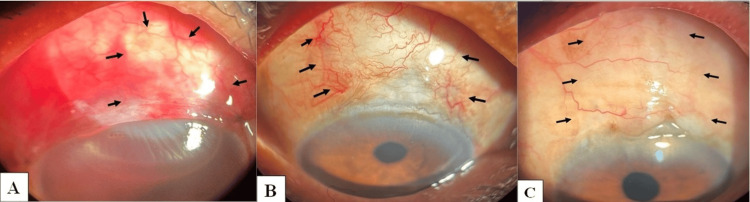
Postoperative photographs depicting bleb morphology (black arrows): (A) first postoperative day image, (B) at one-month follow-up, and (C) at sixth-month follow-up.

Pakravan et al. compared the subtenon injection of MMC with the conventional soaked sponge route and reported blebs with lower height, with less vascularization, and having larger extent in the subtenon injection group at six months follow-up [30]. Gupta et al. compared the subconjunctival injection of MMC at the end of trabeculectomy with the intra-tenon injection of MMC prior to the conjunctival peritomy, and in both groups, they had blebs extending >4 clock hours in 65% eyes and 2-4 clock hours in 35% eyes [27]. In our study also at six months, we observed a diffuse bleb with a larger extent with IBAGS revealing 29 eyes with a high bleb, 29 with a diffuse bleb spanning >2 clock hours, and 25 having low vascularity with no eyes having encapsulated blebs (Table 5; Figure 4). 

A low dose of MMC through the subtenon route, as per our reports, achieved 93.33% complete success and 6.67% qualified success (Table 6). Our reports were much more convincing than the outcomes of Maheshwari et al., who achieved an overall success of 90.5% with 52.4% complete success and 26.1% qualified success in the subtenon injection group, and they compared it with the conventional route using soaked sponges, which achieved an overall success of 87.0% [10]. Lee et al. compared subtenon injection in combined surgery with cataract extraction and trabeculectomy alone and reported a complete success of 86% and 90%, respectively, in both groups [16]. Quist et al. described subtenon injection in patients of trabeculectomy with Ex-PRESS shunt and reported complete success in 60.0% [31].

Rajendrababu et al. reported postoperative complications of a high dose (0.4 mg/ml) of MMC in seven eyes like hyphema, conjunctival buttonhole, conjunctival retraction, aqueous misdirection, and kissing choroid [25]. Maheshwari et al. compared subtenon injection with a soaked sponge approach of 0.2 mg/ml MMC application. They reported nine complications, all in sponges groups, and no significant complications in the injection group [10]. In our study, we observed hypotony in one patient, and six patients had corneal edema, which was well-managed conservatively. It can be explained due to the precise delivery of the desired amount of lower concentration of MMC. 

Our study gives significant results on the outcome of subtenon injection of low-dose MMC in combined trabeculectomy surgery, but it had limitations of a small sample size, and we have not performed a comparative study.

## Conclusions

A low dose of subtenon MMC injection has shown better IOP control, a well-functioning and normally vascularized bleb, and minimal acceptable complications. This approach can be a safe and efficient adjuvant in primary filtering surgeries. It is an effective adjunctive treatment for glaucoma patients at risk of bleb failure, with very few complications associated with the minimal MMC dose. Additionally, it is equally effective in treating both primary open-angle and angle-closure glaucoma. Surgeons may consider MMC subtenon injection as an alternative to the soaked sponge application.
